# Established and new rotavirus vaccines: a comprehensive review for healthcare professionals

**DOI:** 10.1080/21645515.2020.1870395

**Published:** 2021-02-19

**Authors:** Volker Vetter, Robert C. Gardner, Serge Debrus, Bernd Benninghoff, Priya Pereira

**Affiliations:** a Medical Affairs Department, GSK, Wavre, Belgium; b Vaccines R&D – Technical R&D, GSK, Wavre, Belgium

**Keywords:** Rotavirus vaccines, rotavirus vaccine design, coverage, impact

## Abstract

Robust scientific evidence related to two rotavirus (RV) vaccines available worldwide demonstrates their significant impact on RV disease burden. Improving RV vaccination coverage may result in better RV disease control. To make RV vaccination accessible to all eligible children worldwide and improve vaccine effectiveness in high-mortality settings, research into new RV vaccines continues. Although current and in-development RV vaccines differ in vaccine design, their common goal is the reduction of RV disease risk in children <5 years old for whom disease burden is the most significant. Given the range of RV vaccines available, informed decision-making is essential regarding the choice of vaccine for immunization. This review aims to describe the landscape of current and new RV vaccines, providing context for the assessment of their similarities and differences. As data for new vaccines are limited, future investigations will be required to evaluate their performance/added value in a real-world setting.

## PLAIN LANGUAGE SUMMARY

The disease

- Rotaviruses are a leading cause of acute diarrhea, also called gastroenterities, among young children. They can lead to servere dehydration, hospitilization, and even death.
- Several vaccines against rotavirus disease have been developed. Their design is based on:
- weakened human rotavirus that mimic natural infection without causing disease, such as ***Rotarix, Rotavin-M1, Rotavac and RV3-BB*** (not yet marketed)
- non-infective animal viruses such as ***RotaTeq, Rotasiil or LLR***.
- new concepts, such as inactivated vaccines

What is new?

We reviewed the current, recently launched and soon-to-be-launched rotavirus vaccines and found that:

- ***Rotarix*** and ***RotaTeq*** have been used globally for more than a decade with demonstrated impact and favourable safety profile
- Limited data on the impact and safety profile are available to date for:
- ***Rotavin-M1*** and ***LLR vaccines***, locally marketed in Vietnam and China, respectively
- ***Rotavac*** and ***Rotasiil***, licensed in india
- New vaccine concepts have been mainly investigated animal models with encouraging results

What is the impact?

- Despite their different designs, the current rotavirus vaccines demonstrate effectiveness in protecting against rotairus gastroenterits.
- Data for most recent vacciness are currently limited, for which additional data are needed to demonstrate how they will perform on a larger scale, their added value in a real setting and ther safety profile.

## 1 Introduction

Rotavirus (RV) gastroenteritis (RVGE) is a common disease that infects most children before the age of 5 years.^1^^,2^ Developing countries show a higher disease burden compared to developed countries, especially in very young children, due to higher comorbidity rates during childhood and inadequate access to preventive and treatment measures. As a result, the vast majority of RV-associated deaths occur in low-income countries.^2,3^ RV is highly contagious, spreading predominantly through a fecal-oral mode of transmission, and displays resistance to common disinfectants.^4^ Upon ingestion, RVs replicate in the mature villous cells of the small intestine mucosa and cause fever, acute watery diarrhea, and vomiting. The resulting loss of body fluids may lead to severe dehydration, especially in the vulnerable age group of 3 months to 3 years, requiring timely hospitalization and treatment with oral rehydration and/or intravenous fluids.^2,3^ In 2016, RV infection was responsible for an estimated 1,537,000 (95% uncertainty interval [UI], 285,000 − 7,750,500) hospitalizations among children younger than 5 years, globally.^5^

Vaccination has been identified as an efficient strategy to reduce the risk of RV infections and substantially reduce the disease burden. After the first recommendation in 2006, the World Health Organization (WHO) issued a reinforcement in 2009 supporting that RV vaccination should be offered to infants in all regions of the world, especially in regions with high diarrhea-related death rates.^6^ The 2013 WHO position paper on rotavirus vaccines also states that the use of RV vaccines should be part of a comprehensive strategy to control diarrheal diseases using both prevention (e.g. promotion of basic hygienic measures, improved water supply and sanitation) and treatment packages (e.g. oral rehydration therapy).^2^ For more than one decade, RV vaccines have substantially contributed to the global reduction of RV-associated mortality.^5,7^ However, due to unequal coverage of RV vaccines in different regions, RV was still responsible for 128,500 (95% UI, 104,500–155,600) deaths among children younger than 5 years in 2016, nearly all in low- and middle-income countries.^5^

### 1.1 Understanding RV Biology

**Figure 1.**
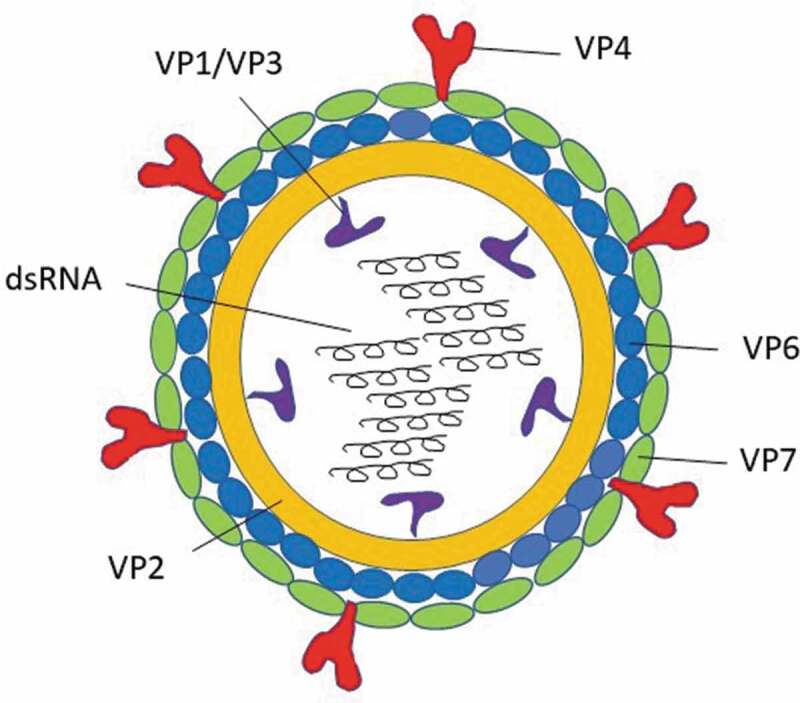
Rotavirus structure and potential vaccine targets.

RNA, ribonucleic acid; VP, structural protein.

RV belongs to the genus *Rotavirus* (family *Reoviridae*) and is a wheel-shaped virus that has three concentric protein layers: an internal capsid (core), an intermediate capsid, and an outer capsid.^8^ Figure 1 presents the structure of RV. The inner core contains the viral genome, which is composed of 11 segments of double-stranded RNA. The 12 proteins encoded by the 11 RNA segments of RV are divided into 6 structural viral proteins (VPs) and 6 non-structural proteins (NSPs).^8^

The amino acid sequence of the structural protein of the inner capsid layer, VP6, is used to classify RV into at least eight groups/species (referred to as RVA-RVH), of which groups A, B, C, and H have been found to infect humans and animals, and group A is the major cause of RV‐associated infections in humans.^9^ Group A RVs are further categorized into genotypes based on differences in the RNA sequences that encode the two external proteins: VP4 and VP7. VP7 (a glycoprotein) determines the genotype G, whereas VP4 (a protease-cleaved protein) determines the genotype P.^8^ More than 90% of group A RV genotypes correspond to one of the following strains: G1P[8], G2P[4], G3P[8], G4P[8], G9P[8], and G12P[8].^2,10^

Several RV proteins are involved in the immune response, including VP4 and VP7, which were shown to induce neutralizing RV-specific antibodies and enhance protective immunity. These proteins along with the highly immunogenic capsid-component VP6 have been crucial in RV vaccine development.^11,12^

However, to date, the immunological correlate of protection for RV remains to be firmly established.^11^

### 1.2 RV vaccine development

Due to the substantial public health burden of RVGE, the need for effective prevention was critical. Following the discovery of RV, research into RV vaccines was initiated.^13,14^ The key observations and requirements for RV vaccine development included:

1. studies showed that a natural, early infection with RV prevented the development of clinically severe forms of the disease upon re-infection, and that repeated exposure to RV induced a broader heterotypic immune response;^12^
2. due to the lack of a definite correlate of protection against RVGE, large efficacy trials to test RV vaccines would need to be based on clinical efficacy endpoints, e.g. moderate-to-severe RV-positive diarrheal disease;^15^ and
3. an ideal RV vaccine should provide early and broad protection against the circulating and evolving RV strains.^16^

These observations led to a first strategy for RV vaccine development using live-attenuated RV, which mimics natural infection and subsequent immune response, but without causing disease. Since interspecies infection is limited (this phenomenon being referred to as “host range restriction”), animal RVs are naturally attenuated for humans. Immunization with animal-based RV vaccines, called the “Jennerian” approach, was tested in the late 1980s but failed to achieve acceptable protection in infants.^17^ Alternative approaches using either attenuated human RV strains or animal-human reassortant RV strains were therefore explored. The production of reassortant vaccines is based on the ability of RVs to combine with each other during mixed infections *in vitro*.^17^ Such a vaccine formulation which consists of some genes from the animal RV parent and from the human RV parent − termed the “modified Jennerian” approach − resulted in the development of the first RV vaccine, Human Rhesus Rotavirus (*HRRV; Rotashield*, Wyeth-Lederle, USA), containing a mixture of four rhesus-human reassortant strains. Licensed in the United States of America (USA) in 1998, *HRRV* was withdrawn approximately one year later due to its association with intussusception (IS), an intestinal invagination that can result in life-threatening bowel obstruction.^18^

Following the withdrawal of *HRRV* and 8 years of further research, two second-generation vaccines reached licensing stages in 2004 and 2006, respectively: 1) *HRV*, an oral human live-attenuated RV vaccine containing a single RV strain (*Rotarix*, GSK, Belgium)^19^ and 2) *HBRV*, an oral bovine-human reassortant vaccine containing five reassortant strains (*RotaTeq*, Merck & Co., Inc, USA).^20^ Both candidate vaccines published their landmark phase III trial results in the same issue of *The New England Journal of Medicine* in January 2006.^21,22^ They have since become the two most commonly used RV vaccines worldwide.^23^ Their routine use in the National Immunization Program (NIP) is recommended by several national health authorities,^23^ including the CDC (Centers for Disease Control and Prevention) in the USA,^24^ where these two vaccines are available for use. Although differing in concept, *HRV* and *HBRV* have both had a tremendous impact on the burden of RV disease.^14^

Other RV vaccines − following similar vaccine concepts to those employed for the two widely established vaccines − are either already locally marketed or in various development stages. Although several options are already available, research and development of new RV vaccines is ongoing with the aim of improving the global supply of RV vaccines, reducing vaccine cost, and improving vaccine effectiveness, in particular in developing countries.^17,25^ The early phase vaccines (pre-clinical stage) that are currently being developed provide novel approaches to promoting anti-RV immunity, such as inactivated virus, expression of viral recombinant proteins, or virus-like particles (VLPs).^25,26^ Thus, a unique vaccine landscape comprising several vaccine concepts is emerging in the field of protection against RV.

The different concepts of RV vaccines may result in different vaccine properties. Nevertheless, as licensed RV vaccines are able to provide broad protection against a variety of non-vaccine type RV strains,^27–29^ the choice between these is often driven by programmatic considerations. To make the most appropriate choice for the implementation of RV vaccination, it is crucial for health authorities, healthcare practitioners, and other public health experts to understand the rationale, advantages, and limitations of the different RV vaccine options. While scientific evaluation of the currently available vaccines is well covered by published literature, our contribution emphasizes selection in private market in clinic use and programmatic issues in universal mass vaccination use, which could impact preferences among practitioners and recommending bodies. This literature review was conducted to gather and compare currently available information for established, recently licensed and in-development RV vaccines.

## 2 Methodology

This article is a non-systematic, comprehensive literature search carried out between the 1^st^ of February 2017 and the 31^st^ of August 2019 in PubMed and Embase with the aim of mapping the characteristics of RV vaccines (pre- and post-registration/licensure) according to their vaccine design, immunogenicity, efficacy, effectiveness, impact, and safety data for marketed and in-development RV vaccines. We subdivided the overall search into individual searches for each RV vaccine, with search terms including brand name, generic name, and manufacturer of the vaccine.

We complemented the literature search with a parallel search on Google and Google Scholar using the search terms mentioned above. The Google internet search enabled us to retrieve conference presentations and result summaries of products in clinical development or early stages of market launch for which the published data from journal articles retrieved via PubMed was limited. Additional sources of summarized data for the well-established *HRV* and *HBRV* vaccines, such as the summaries of product characteristics, were also identified through a Google search.

Among the results obtained, we considered information sources and articles with abstract and/or full text written in English. For the extensively studied *HRV* and *HBRV* vaccines, we selected recent informative articles that contained summarized data. For vaccines in late-stage development and locally marketed vaccines (including those recently prequalified by the WHO), we took into consideration all sources retrieved from PubMed, Google, Google Scholar, and Embase searches.

## 3 Results

The results of the literature search enabled us to gather the characteristics of the different RV vaccines including strains used, dosage, and presentation, clinical data on vaccine efficacy, effectiveness, and safety. We classified the vaccines and presented their data in tables as follows:

-Table 1: oral, live-attenuated, single-strain vaccines based on human RV strains (including neonatal strains): *HRV (Rotarix), Rotavin-M1, 116E (Rotavac*, a naturally occurring human-bovine reassortant) and *RV3-BB*;

**Table 1.** Characteristics of rotavirus single-strain vaccines based on neonatal or live-attenuated human RV strains

	*HRV (Rotarix)*	*Rotavin-M1*	116E (Rotavac)*	*RV3-BB* (in development)
Basic concept (composition/strain)	Human, live-attenuated G1P [8] RV vaccine.^19^	Human, live-attenuated G1P[8] RV vaccine.^30^	Live-attenuated, naturally reassorted human-bovine single strain G9P[11] vaccine, containing one bovine RV gene P[11] and 10 human RV genes including G9.^29,31^	Human, neonatal G3P[6] RV vaccine.^32^
Manufacturer, country, licensure and WHO prequalification	GSK; first approved in Mexico in 2004, Europe in 2006 and USA in 2008(WHO-prequalified vaccine).^19^	Center for Research and Production of Vaccines and Biologicals (Polyvac); national license granted by Vietnam in 2012.^30,33^	Bharat Biotech Int. Ltd and Department of Biotechnology, Government of India; licensed in India in 2014. (WHO-prequalified vaccine).^34,35^	Murdoch Children’s Research Institute/Biofarma; phase III development in Australia, New Zealand and Indonesia.^32^
Dosage and schedule, presentation, and shelf life	2 oral doses: first dose administered from age 6 weeks. At least 4 weeks between doses. Course must be preferably completed by 16 weeks and always before 24 weeks of age.^19^ Liquid: either oral applicator or oral tube; shelf life of 3 years at 2–8°C.Lyophilized: oral applicator; shelf life of 3 years at 2–8°C.^19^	2 oral doses administered 2 months apart. The first dose must be administered at 6–12 weeks of age.^33^ Liquid; 24 months at −20°C 2 months from thawing at 5°C.^33^	3 doses at 6, 10, and 14 weeks of age. Oral liquid, frozen; shelf life of 5 years at −20°C or 6 months at 5 ± 3°C. Fully liquid also available.^31^	3 oral doses administered as either a neonatal schedule (0–5 days, 8 weeks, and 15 weeks of age) or an infant schedule (8, 15, and 24 weeks of age). Liquid; n published data on storage conditions.^32^
Protection against non-vaccine strains	Broad protection demonstrated.^19^	No published results.	Efficacy against heterotypic G1P[8], G2P[4], G12P[6], and G12P[8] RV strains and partially heterotypic G9P[4] strain demonstrated in a phase III trial.^29,31^	No published results.
Vaccine efficacy, %; and/or immunogenicity,%	*Low-income African and Asian countries*: 61.2%–100% (severe EP)^19^ *High-income and upper-middle-income countries*: 95.8% (severe EP)^19^ *LATAM countries*: Efficacy against RVGE-associated hospitalizations/emergency visits: 83%–85%.^19,36^	*Vaccine immunogenicity (as RV IgA) seroconversion)*:^30^Higher titer (10^6.3^), 2-dose regimen: 73%Lower titer (10^6.0^), 2-dose regimen: 61%.	*Efficacy against severe RVGE* *(score ≥11/20)*:^29,31^ In the first year of life: 56% [95% CI: 37–70]. In the second year of life: 49% [95% CI: 17–68].	*Cumulative vaccine take after 3 doses*:^32^Neonatal schedule: 90%Infant schedule: 93%. *Vaccine efficacy against severe RVGE in Indonesia*:^37^ Neonatal schedule: 75% [95% CI: 44–91]Infant schedule: 51% [95% CI: 7–76]
Vaccine effectiveness, %	Effectiveness against combined RV hospitalizations, emergency visits, and outpatient visits:^38,39^*High-income (low child mortality) countries*: 81%–84%*Upper-middle-income (medium child mortality) countries*: 54%–75%*Lower-middle-income (high child mortality) countries*: 63% – 57%	No published results.	No published results.	No published results.
Safety	Well characterized safety profile Large post-marketing studies Benefit-risk ratio positive Continuous safety monitoring^20^	Acceptable safety profile in one clinical trial (with limited sample size).^30^	Acceptable safety profile in a few clinical trials (with limited sample size). Recent implementation of nationwide safety surveillance^40^	Acceptable safety profile in a few clinical trials (with limited sample size).^32^

*Naturally occurring human-bovine reassortant; CI, confidence interval; EP, endpoint; IgA, immunoglobulin A; LATAM, Latin America; RV, rotavirus; RVGE, rotavirus gastroenteritis.

-Table 2: oral, live-attenuated, single-strain vaccine based on animal RV strains: *Lanzhou lamb rotavirus vaccine (LLR)*;

**Table 2.** Characteristics of rotavirus single-strain vaccines based on live-attenuated animal RV strains

	*LLR* (Lanzhou lamb rotavirus vaccine)
Basic concept (composition/strain)	Lamb, live-attenuated G10P[15] RV vaccine^41^
Manufacturer, country, licensure and WHO prequalification	Lanzhou Institute of Biomedical Products(China National Biotec Group [CNBG]).National license granted by China in 2000.^41^
Dosage and schedule, presentation, and shelf life	3 oral doses: 1 dose per year for 3 consecutive years in children aged 2–36 months. Liquid; shelf life of 1 year at 2–8°C.^33,42^
Protection against non-vaccine strains	No published results.
Vaccine efficacy, %	No published results.
Vaccine effectiveness, %	*1 dose of LLR compared with no vaccination*:^41,43^Effectiveness among children <5 years of age: 35% [95% CI: 13–52]; 52% against G3 [95% CI: 2–76].^44^Effectiveness among children <24 months of age: 77% [95% CI: 64–86].^43^
Safety	No published data in English language.

RV, rotavirus; RVGE, rotavirus gastroenteritis; CI, confidence interval.

-Table 3: oral, live-attenuated, single- or multi-strain vaccines based on animal-human reassortant RV strains: *HBRV (RotaTeq), Bovine Rotavirus Pentavalent BRV-PV (Rotasiil), tetravalent UK-BRV, hexavalent UK-BRV*, and *pentavalent UK-BRV*;

**Table 3.** Characteristics of rotavirus multi-strain vaccines based on live-attenuated bovine-human reassortant RV strains

	HBRV (RotaTeq)	BRV-PV (Rotasiil)	Tetravalent UK-BRV	Hexavalent UK-BRV	Pentavalent UK-BRV
Basic concept (composition/strain)	Live-attenuated, human-bovine reassortant vaccine: 5 reassortant strains in one vaccine containing human G1, G2, G3, G4 (VP7) and P[8] (VP4) inserted into the bovine G6P[5].^20^	Live-attenuated, human-bovine reassortant vaccine: 5 reassortant strains in one vaccine containing human G1, G2, G3, G4 and G9 (VP7) inserted into the bovine G6P[5] UK strain.^45,46^	Live-attenuated, human-bovine reassortant vaccine: 4 reassortant strains including human G1–4 inserted into the bovine G6P[5] UK strain.	Live-attenuated, human-bovine reassortant vaccine: 6 reassortant strains including human G1–4, G8, G9 inserted into the bovine G6P[5] UK strain.^47^	Live-attenuated, human-bovine reassortant vaccine: 5 reassortant strains including human G1–4, G9 inserted into the bovine G6P[5] UK strain
Manufacturer, country, licensure and WHO prequalification	Merck & Co, Inc (MSD); licensed by FDA and EMA in 2006(WHO-prequalified vaccine).	Serum Institute of India Limited; licensed in India in 2017. (WHO-prequalified vaccine).	Developer: Shantha Biotechnics (phase I/II development) – development abandoned.	Developer: Wuhan Institute of Biological Products, China. Development phase: phase I, age-descending study completed in China (ChiCTR-PIR-16,008,824).^47^	Developer: Instituto Butantan, Brazil. Development phase: phase I study in Brazil.^48^
Dosage and schedule, presentation, and shelf life	3 doses administered ≥4 weeks apart; the 3-dose course should be completed by 20–22 weeks of age and not later than 32 weeks of age. The first dose is administered between 6–12 weeks of age. Oral, liquid, squeezable tube;shelf life of 2 years at 2–8°C.	3 doses at 6, 10, and 14 weeks of age.^49^ Oral, lyophilized product to be reconstituted before administration; shelf life of 36 months below 25°C, 18 months between 37°C and 40°C and short time periods over 55°C.^50^	3 doses: initial dose at 6–8 weeks and following doses at 4-week intervals.Oral, liquid, ready to administer.	No information available.	No information available.
Protection against non-vaccine strains	Broad protection largely demonstrated.^20,28^	No published results.	No published results.	No published results (early phase clinical development).	No published results (early phase clinical development).
Vaccine efficacy, % [95% CI] and/or immunogenicity,%	*Low-income African and Asian countries*:^51–53^ 34%–87.5%.*High-income and upper-middle-income countries*:^21,22,54,55^ 85%–98%.*LATAM countries*:^36^Efficacy against RVGE-associated hospitalizations/emergency visits: 90%.	*Efficacy against RVGE in Niger*:^45^Severe cases (per-protocol analysis): 67% [50–78]. *Efficacy against RVGE in India*:^49^Severe cases (per-protocol analysis): 36% [12–54]Very severe cases: 61% [18–81]. *Anti-RV IgA seroconversion after 3 doses*:^46^ 60%.	A phase I/II study conducted in India showed seroresponse rates up to 83.3% depending on the viral titer.^56^ Non-inferiority over HBRV non demonstrated.^57^	No information available.	A phase I study in Brazil showed good safety profile and evidence of immunogenicity in adults.^48^
Vaccine effectiveness, % [95% CI]	Effectiveness against combined RV hospitalizations, emergency visits, and outpatient visits.^39^*High-income (low child mortality) countries*: 90%*Lower-middle-income (high child mortality) countries*: 45%	No published results.	No information available.	No information available.	No information available.
Safety	Well characterized safety profile. Large post-marketing studies. Benefit/risk ratio largely positive. Continuous safety monitoring.^58^	Acceptable safety profile in a few clinical trials (with limited sample size).	Acceptable safety profile in one clinical trial (with limited sample size).	No published results (early phase clinical development).	Acceptable safety profile in one clinical trial (with limited sample size).

BRV-TV G1–4 tetravalent candidate (Shantha Biotechnics, India); CI, confidence interval; EMA, European Medicines Agency; FDA Food and Drug Administration; G1–4, 8, 9-hexavalent candidate, human-bovine reassortant hexavalent RV vaccine (Wuhan Institute of Biological Products, China); G1–4, 9-pentavalent candidate, human-bovine reassortant pentavalent RV vaccine (Instituto Butantan, Brazil); IgA, immunoglobulin A; LATAM, Latin America; RV, rotavirus; RVGE, rotavirus gastroenteritis.

-Table 4: parenteral, non-live vaccines in early phase of development: *inactivated RV vaccine, recombinant proteins*, and *VLPs*.

**Table 4.** Characteristics of new rotavirus vaccines in early phase development

	*G1P*[8], inactivated	*P2-VP8-P*[8] and *P2-VP8-P*[4/6/8]	*MBP::VP6*and *pCWA:VP6*	*VP2/6/7*and*VP2/4/6/7VLPs*	*VP6 GI.3/GII.4 RV-NoV VLPs*
Basic concept	Inactivated human RV vaccine.	Subunit RV vaccines based on recombinant proteins.		Subunit RV vaccines based on virus-like particles.	
Composition	Thermally inactivated G1P[8] strain derived from a fecal specimen of a child in USA.	Monovalent P2-VP8-P[8]: 1 truncated human VP8 protein (P[8]) and the P2 epitope of the tetanus toxin. TrivalentP2-VP8-P[4/6/8]: 3 truncated human VP8 proteins (P[4], P[6], P[8]) and the P2 epitope of the tetanus toxin.	Recombinant murine VP6 protein expressed as chimera with MBP, and VP6-CWA protein expressed on the surface of *L. lactis.*	RV-VLPs: co-expression of VP2, VP6, VP4 and/or VP7 (expression systems using baculovirus, *E.coli*, plant or yeast).	Recombinant human RV VP6 protein combined with NoV VLPs.
Development phase and developer	Preclinical development.^59,60^Centers for Disease Control and Prevention (CDC), USA.	Clinical development.^61,62^ PATH Rotavirus Vaccine Program, USA.	Preclinical development.Cincinnati Children’s Hospital, USA; Laboratoria de Immunologia y Virologia (LIV), Argentina.	Preclinical development.Baylor College of Medicine, USA.	Preclinical development.University of Tampere School of Medicine, Finland.
Route of administration tested	Parenteral.	Parenteral.	Parenteral.	Parenteral.	Parenteral.
Results obtained	Three doses induced high titers of RV-specific IgG antibodies and heterotypic immunity in gnotobiotic piglets and guinea pigs.^59,60^Mucosal immunity in the gut and strong serum immune response shown in mice.^63,64^	Monovalent P2-VP8-P[8]: It was well tolerated and evoked immune responses when intramuscularly injected in healthy adults in USA^61^ and in South African toddlers and infants.^62^ Trivalent P2-VP8-P[4/6/8]: phase I/II trial completed in South Africa (NCT02646891) but results not available.	Intranasal administration of MBP::VP6 (together with an adjuvant) to mice conferred 98% protection against infection by a murine RV strain.^65^Humoral response of mice immunized with recombinant VP6 protein expressed on *L. lactis* proved to be immunogenic.^66,67^	RV VLPs induced significant immune response independent of the immune route (intramuscular, intrarectal, intranasal, intraperitoneal or oral) in mice.^68,69^	When delivered intranasally or intramuscularly to mice, this candidate vaccine was immunogenic and conferred protection against infection by a murine RV^70–72^
Additional information	Potential lower risk of IS. No risk of vaccine-derived reassortant strains. Inactivated vaccines are generally less efficacious than live vaccines.	Potential lower risk of IS. Non-oral RV vaccines could present higher efficacy in developing countries compared to oral RV vaccines. No risk of vaccine-derived reassortant strains. Potential protection against NoV and RV gastroenteritisin a single vaccine (for RV-NoV VLPs).VLPs are generally more immunogenic than subunit or recombinant immunogens.^68^Possible combination of an oral attenuated HRV vaccine with RV VLPs (avoiding the need of sequential doses with live vaccine).^73^			

G1P[8], inactivated human RV vaccine (Centers for Disease Control and Prevention, USA); P2-VP8-P[8], monovalent subunit vaccine P2-VP8-P[8] (PATH Rotavirus Vaccine Program, USA); P2-VP8-P[4/6/8], trivalent subunit vaccine P2-VP8-P[4]P[6]P[8] (PATH Rotavirus Vaccine Program, USA); MBP::VP6, maltose-binding protein-VP6 protein chimera (Cincinnati Children’s Hospital, USA); VP2/6/7 VLPs (trivalent) and VP2/4/6/7 VLPs (quadrivalent), virus-like particles RV vaccine (Baylor College of Medicine, USA); VP6-GI.3/GII.4 RV-NoV VLPs, rotavirus-norovirus virus-like particles combination vaccine (University of Tampere School of Medicine, Finland).CWA, cell wall anchor; IgG, immunoglobulin G; IS, intussusception; MBP, maltose-binding protein; NoV, norovirus; RV, rotavirus; USA, United States of America; VLPs, virus-like particles PATH, Program for Appropriate Technology in Health.

**Figure 2.**
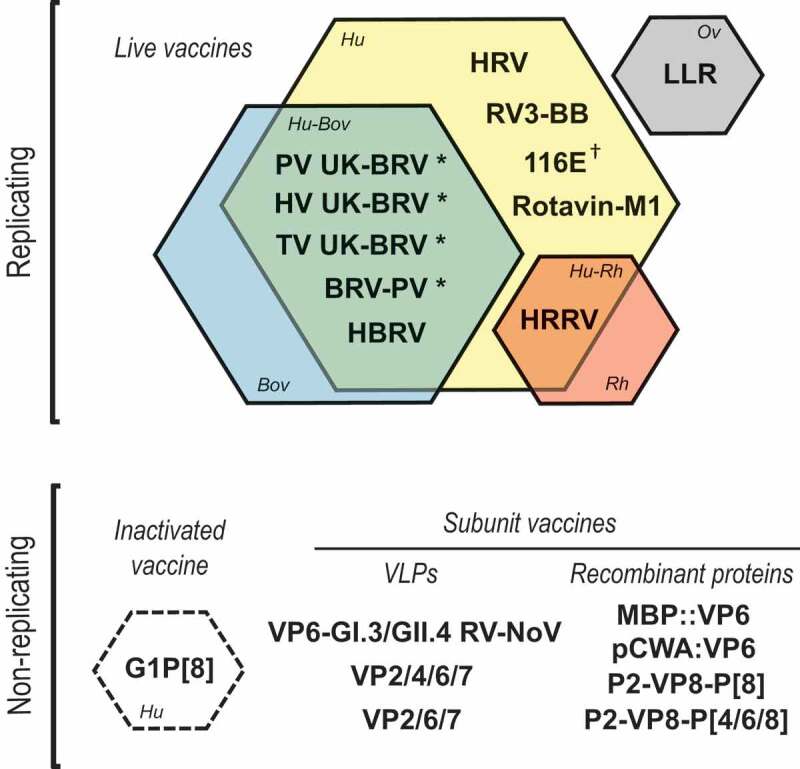
Overview of the rotavirus vaccines included in this review reflecting their vaccine concept and type of strain.

Figure 2 illustrates the landscape of RV vaccines included in this review, specifically vaccine concept and type of strain.

RV, rotavirus; NoV, norovirus; Ov, ovine RV; Bov, bovine RV; Hu, human RV; Rh, rhesus RV; Hu-Bov, human-bovine reassortant RV; Hu-Rh, human-rhesus reassortant RV; VLPs, virus-like particles.* Vaccines derived from the bovine G6P[5] UK-BRV strain.† Human strain with a single gene segment encoding VP4 derived from a bovine rotavirus (natural bovine-human reassortant)Live vaccines (clockwise): HRV (Rotarix, GSK, Belgium); LLR (Lanzhou lamb RV vaccine, Lanzhou Institute of Biomedical Products, China); RV3-BB (human neonatal RV vaccine, Bio Farma Indonesia, Indonesia, and Murdoch Children’s Research Institute of Australia, Australia); 116E (Rotavac, Bharat Biotech, India); Rotavin-M1 (Polyvac, Vietnam); HRRV, human-rhesus reassortant RV vaccine (Wyeth, USA); HBRV (RotaTeq, Merck & Co., Inc, USA); BRV-PV (Rotasiil, Serum Institute of India, India); tetravalent UK-BRV (Shantha Biotechnics, India); hexavalent UK-BRV (Wuhan Institute of Biological Products, China); pentavalent UK-BRV (Instituto Butantan, Brazil).Inactivated vaccine: G1P[8], human inactivated RV vaccine (Centers for Disease Control and Prevention, USA).Subunit vaccines based on virus-like particles (VLPs): VP6-GI.3/GII.4, trivalent, RV-NoV virus-like particles combination vaccine (University of Tampere School of Medicine, Finland). VP2/4/6/7 RV quadrivalent, virus-like particles RV vaccine (Baylor College of Medicine, USA); VP2/6/7, trivalent, virus-like particles RV vaccine (Baylor College of Medicine, USA).Subunit vaccines based on recombinant proteins: MBP::VP6 and pCWA:VP6, maltose-binding protein-VP6 protein chimera and VP6-CWA fusion protein (Cincinnati Children’s Hospital, USA; Laboratorio de Immunologia y Virologia (LIV), Argentina); P2-VP8-P[8], monovalent P2-VP8 RV vaccine (PATH Rotavirus Vaccine Program, USA); P2-VP8-P[4/6/8], trivalent P2-VP8 RV vaccine (PATH Rotavirus Vaccine Program, USA).

## 4 Discussion

A great variety of RV vaccines, using different approaches and concepts, have emerged over the last two decades with the common goal of preventing RVGE. As presented in the results section of this literature search (Tables 1–table 4), each vaccine concept and every vaccine within each concept show different biological properties (e.g. strain type and virus concentration) that may translate into different vaccine characteristics (e.g. dosing schedule, efficacy, effectiveness, and safety profile). A solid understanding of RV vaccine characteristics is therefore essential to support informed health policy decisions in individual countries and clinical practice.

### 4.1 Vaccine design – oral, live-attenuated, single- and multi-strains

RV vaccines can be of human or animal-human reassortant RV strain origin. In addition, the vaccine can be single-strain (if derived from a single RV strain) or multi-strain (if several strains are combined within a single vaccine). While vaccines designed on single live-attenuated RV strains are based on the observation that a first contact with RV prevents clinical symptoms in subsequent re-infections with the same or different wild-type strains, multi-strain reassorted RV vaccines’ design suggest that exposure to different strains of RV may confer protection against a broad range of circulating RV strains.^12,16,17^ In this regard, both clinical data and real-world evidence derived from global studies with well-established vaccines have shown that both multi-strain (*HBRV*) and single-strain (*HRV*) RV vaccines can protect against several strains of RV.^27,28^ This broad protection is particularly relevant because RV strain distribution displays seasonal and geographical variations.^74,75^

Of note, the temporary predominance of the G2P4 strain in countries with high RV vaccination coverage (especially for *Rotarix*) − in the context of a substantial overall high vaccination coverage-related decrease in RV cases − has prompted scientific discussions regarding its potential emergence due to a link between RV vaccination and the prevalence of non-RV vaccine strains.^76,77^ However, data gathered so far from global RV surveillance networks support the occurrence of a natural cycling in RV strain distribution and dominant types. Moreover, similar unpredictable changes in strain fluctuations have been reported both in countries with and without routine RV vaccination.^78–80^ There is therefore a lack of substantial evidence supporting the hypothesis of a shift in RV strain distribution driven by vaccine-induced pressure, and it seems likely that these changes reflect natural fluctuations in RV strains in time and space.^28,81^ Although these data are reassuring with regards to the circulation of non-vaccine RV strains, the risk of appearance of new RV strains arising from the segmented genome of RV is a strong argument for a continued and efficient epidemiological surveillance of RV strains.^82^

#### 4.1.1 Single-strain vaccines – neonatal naturally-occurring RV strains

Neonatal strains of RV appear to be naturally attenuated: it was observed that asymptomatically infected neonates had a reduced frequency and severity of RV-associated diarrhea in subsequent RV infections. This led to the exploration of RV neonatal strains as vaccine candidates.^17^

##### Rotavac; a naturally occurring human-bovine reassortant neonatal strain

In the early 2000s, two RV strains obtained from asymptomatic-infected newborns in Delhi (strain *116E*) and Bangalore (strain *I321*) were tested as vaccine candidates in India. Each strain appeared to be a naturally occurring human-bovine reassortant: *116E* (genotype G9P[11]) is a human RV strain bearing a single gene segment derived from a bovine RV, while *I321* (genotype G10P[11]) is a bovine strain with two gene segments derived from a human RV. In a phase I trial, *116E* was able to induce a superior immune response compared to *I321* and placebo, and was hence selected for further development.^83^

In 2011, a phase III randomized, double-blind, placebo-controlled trial in India evaluated the safety and efficacy of 3 doses of *116E* administered at 6, 10, and 14 weeks of age in more than 6,500 infants. The estimated efficacy of *116E* against severe RVGE requiring hospitalization or supervised rehydration was 56% (95% confidence interval [CI] [37%–70%]) in the first year of life and 49% (95% CI [17%–68%]) in the second year of life (Table 1).^29,31^ The occurrence of adverse events was not significantly higher in the vaccine group compared to the placebo group; however, the study had insufficient power to conclude on the occurrence of IS between both groups.

Another study showed no interference of *116E* in the immune response of co-administered vaccines routinely included in NIPs, such as *oral polio vaccines* (*OPV*) or *pentavalent vaccines* against diphtheria, pertussis, tetanus, hepatitis B, and *Haemophilus influenzae* type b.^34^ The *116E* vaccine was licensed in 2014 in India and recently obtained WHO prequalification.^35^

##### RV3-BB (naturally attenuated human neonatal strain vaccine; in development)

The human neonatal G3P[6] strain RV3-BB, developed by Murdoch Children’s Research Institute in Australia, was found to replicate well in the newborn gut and to provide protection from severe RVGE.^84^ These findings have led to a birth dose strategy with the goal of providing early protection against RV. After conclusive phase I results, a phase IIa trial reported *RV3-BB* to be immunogenic and well tolerated when given according to a 3-dose neonatal or infant schedule (Table 1).^32,85^ More recently, in a phase IIb, randomized, placebo-controlled trial in Indonesia conducted between 2013 and 2016, the vaccine efficacy against severe RVGE was 75% (95% CI [44%–91%]) for infants receiving the neonatal schedule (0 to 5 days, 8 weeks and 14 weeks of age), and 51% (7%–76%) for infants receiving the infant schedule (8 weeks, 14 weeks and 18 weeks of age).^37^ In this study, a similar safety profile was observed across the neonatal schedule, infant schedule and placebo groups. A phase II dose-ranging study is ongoing in African neonates and infants.^86^

#### 4.1.2 Single-strain vaccines − human RV strain

##### HRV (Rotarix)

*Rotarix (HRV)* is a human RV vaccine composed of a G1P[8] strain obtained from the stool of an infant who experienced natural RV infection in the 1988–1989 RV season in the USA.^12^ This vaccine was observed to provide protection against severe infections in subsequent RV seasons. The virus was attenuated by cell culture passages, and the final vaccine − obtained at GSK − underwent robust worldwide clinical development (*RIX4414, HRV, Rotarix*) (Table 1). *HRV* was first registered in Mexico in 2004 and its use has been characterized by extensive post-marketing studies to document safety, effectiveness, and impact.^17,38,87^ *HRV* is currently registered worldwide in >100 countries and is WHO prequalified.^35,88^ The vaccine is administered in 2 doses between the ages of 6 and 24 weeks.^19^

##### Rotavin-M1

*Rotavin-M1* is a frozen oral vaccine containing a G1P[8] strain obtained from a Vietnamese child (Table 1). A dose-escalation study was primarily carried out in a small sample of Vietnamese infants to determine the vaccine dose and schedule. The vaccine formulation eliciting the highest immunoglobulin (Ig)A seroconversion rate (73%, 95%CI [58%‒88%]) was also shown to be well tolerated.^30^ Although no efficacy results were released, the vaccine was licensed in Vietnam in 2012 based on the immunogenicity data. Since then, the vaccine has only been available on the private market with a 2-dose schedule at 2 and 4 months of age. However, the vaccine is currently being introduced into the Expanded Program on Immunization schedule of selected Vietnamese districts on a pilot basis.^26^ A phase III immunogenicity trial of a liquid, nonfrozen formulation of the vaccine is also being planned.^25,26,30,89^

#### 4.1.3 Single-strain RV Vaccines – animal strains

##### Lanzhou lamb rotavirus vaccine (LLR; lamb-derived RV vaccine)

*LLR* is a single RV strain G10P[15], lamb-derived, 3-dose vaccine developed and produced by the Lanzhou Institute of Biological Products (Table 2).^90^ The vaccine is licensed in China since 2000, but since it is not part of a nationally funded program, the coverage is relatively low and geographically variable. The dosing schedule is one dose per year from age of 2 months to 3 years.^91^ Since, to date, no placebo-controlled phase III trial has been conducted, few data are available on the vaccine’s safety, immunogenicity, and efficacy. However, estimates for vaccine effectiveness against RVGE have been provided by several case-control studies, ranging from 35% to 77%.^41–44^ A recent ecological study conducted during nine seasons revealed an inverse relationship between vaccination coverage and RVGE incidence.^91^

#### 4.1.4 Multiple-strain RV Vaccines – licensed bovine-human reassortant

##### HBRV (RotaTeq; pentavalent, bovine-human reassortant, live-attenuated)

*RotaTeq (HBRV)* is a multi-strain bovine-human reassortant (WC3), developed by Merck & Co, Inc. (Table 3). Four reassortant RVs express the VP7 protein (G1, G2, G3, or G4) from the human RV parent strain, and the VP4 protein (P[5]) from the bovine RV parent strain. The fifth reassortant RV contained in the vaccine expresses the VP4 protein (P[8]) from the human RV parent strain and the outer capsid protein (G6) from the bovine RV parent strain.^20^ As was the case for *HRV, HBRV* underwent extensive worldwide clinical development followed by large post-licensure studies reporting its positive impact and safety profile.^20,39,87^ *HBRV* was licensed in February 2006 by the USA Food and Drug Administration and its administration is routinely recommended according to a 3-dose oral schedule at 2, 4, and 6 months of age. The first dose should be given between 6 and 12 weeks of age, with the two subsequent doses administered at 4–to–10-week intervals before the child reaches the age of 32 weeks.^20^ Similar to *HRV, HBRV* is registered worldwide in >100 countries and has been prequalified by the WHO.^35,92^

##### BRV-PV (Rotasiil; pentavalent, bovine-human reassortant, live-attenuated)

*Rotasiil (BRV-PV)* is a multi-strain bovine-human reassortant vaccine containing genotypes G1, G2, G3, G4, and G9 (Table 3). The lyophilized presentation is a thermostable vaccine and retains its stability at temperatures up to 25°C for up to 36 months, between 37°C and 40°C for 18 months, and for short time periods over 55°C.^50^ Two randomized, double-blind, placebo-controlled trials were conducted in Niger and India to evaluate its efficacy. In both trials, healthy infants received three doses of the vaccine or placebo at 6, 10, and 14 weeks of age, along with routine vaccines. The primary efficacy analysis against severe RVGE highlighted a vaccine efficacy of 67% (95% CI [50%–78%]) in Niger and 36% (95% CI [12%–54%]) in India.^45,49^ Although there was no imbalance in the risk of adverse events across vaccine and placebo groups, these two studies were not powered to detect an increased incidence of rare events, such as IS. A study conducted in India reported that the vaccine does not interfere with the immunogenicity of concomitantly-administered routine pediatric vaccines.^93^ A liquid formulation of *BRV-PV* has been developed and recently proved to be non-inferior to the lyophilized formulation.^94^ The *BRV-PV* vaccine was licensed in 2017 in India and recently obtained WHO prequalification.^35^

##### Multiple-strain RV Vaccines – additional bovine-human reassortant under clinical development

###### Tetravalent UK-BRV (bovine-human reassortant vaccine candidate)

This vaccine candidate, whose development was initiated by Shantha Biotechnics (India), contains RV strains with VP7 genotypes G1, G2, G3, and G4 (Table 3). Its safety and immunogenicity were evaluated in phase I and II studies, in which IgA seroconversion rates for the two highest vaccine titers (10^5.8^ and 10^6.4^ focus forming units [FFU]/mL) after administration of three doses ranged from 52.9% to 83.3%.^56^ A phase III clinical trial was conducted involving 1,200 Indian infants aged 6–8-weeks to show non-inferiority against a currently licensed vaccine based on immunogenicity, but the study failed to achieve its main endpoint.^57^ The development of this vaccine is not being pursued any further.^25^

###### Pentavalent UK-BRV (bovine-human reassortant vaccine candidate)

Butantan Institute (Brazil) developed a vaccine candidate that contains RV strains with VP7 antigens G1, G2, G3, G4, and G9 (Table 3). This vaccine was found to be safe and immunogenic in a phase I trial conducted on 79 adult males.^48^ However, the vaccine development has been hampered by difficulties to conduct further clinical trials in Brazil, where routinely used *HRV* has already demonstrated significant benefits with regards to the disease burden.^25^

###### Hexavalent UK-BRV (bovine-human reassortant vaccine candidate)

This hexavalent vaccine candidate, currently in development at the Wuhan Institute of Biological Products (WIBP; China), contains the six reassortants developed by the National Institutes of Health (NIH), i.e. G1–4, G8, and G9 (Table 3).^47^ Although a phase I safety trial has been initiated in 2016, no results have been publicly reported yet.^25,33^

*Multiple-Strain RV Vaccines – Additional Lamb-Human Reassortant under Clinical Development (trivalent lamb-derived RV vaccine)*

This trivalent genetic reassortant vaccine candidate developed at the WIBP, China, uses the lamb strain from the *LLR* as a backbone and contains the VP7 antigens G2, G3, and G4. A phase III trial has been underway since 2016 with planned disclosure of results in 2020.^25,33^

### 4.2 Vaccine design – parenteral, non-live, RV vaccines

Oral RV vaccines, although successful, display a reduced vaccine efficacy in low-income countries, that hinder their fulfillment worldwide.^95^ This lower vaccine efficacy is thought to be associated − among other factors − with characteristics of the intestinal tract, including gut microbiota, maternal antibodies, and enteric co-infections, that are known to display population-specific variations.^92^ By bypassing potential interferences with enteric environment, parenteral vaccines could offer a solution to overcoming the variable levels of vaccine efficacy observed in different target populations. In addition, due to their nature and mode of administration, these vaccines may eliminate risks of vaccine-associated increased IS risk. Moreover, parenteral vaccines have the added benefits that they can be used in combination with other injectable pediatric vaccines and can be produced at relatively low cost.^25,26^

A parenteral inactivated RV vaccine based on a G1P[8] strain is under development and has been tested in animal models with proven efficacy and heterotypic antibody response (Table 4).^59,60^ An alternative delivery approach using microneedles was also evaluated in mice and piglets.^63,64^ This preparation is also being considered for use as a combination vaccine with inactivated polio vaccine, and has proven to have no interference in the immune response to either component in mice studies.^25,26,96^

The subunit vaccine *P2-VP8-P*[8] (Table 4) is a parenteral RV vaccine candidate and consists of a truncated VP8 subunit of the rotavirus Wa strain G1P[8] fused with the P2 epitope from tetanus toxin (Table 4). After safety was demonstrated in adults,^61^ the vaccine was assessed in South African toddlers and infants where it was found to be well tolerated, and displayed a strong IgG response (>98% seroconversion in vaccinated infants compared to 9% in infants receiving placebo).^62^ These study results constituted the basis for a phase I/II trial aiming to assess the safety and immunogenicity of a trivalent subunit vaccine (P2-VP8-P[4]P[6]P[8]) in South African cohorts – the vaccine was shown to be well tolerated with promising anti-P2-VP8 IgG and neutralizing antibody responses among the three vaccine P types.^97^ A multinational phase IIb/III efficacy trial with active comparator for prevention of severe gastroenteritis in healthy infants is currently under way (NCT04010448).^98^

Research has also focused on the VP6 protein since it appears to be the most immunogenic and highly conserved protein in Group A RV (Table 4).^65^ The VP6 subunit vaccine stemming from this research was shown to induce RV-specific antibodies and to prevent viral infection in a murine model of rotavirus infection.^66,67^ Finally, RV-like particles offer a new approach (RV-VLPs) for the development of a subunit RV vaccine. Similarly, a VP6 subunit vaccine, designed as a combination vaccine that incorporates norovirus VLPs (Table 4), has already been shown to elicit satisfactory immune responses in mice.^70^

### 4.3 Dosing schedules

One notable difference between the vaccines reviewed in this article is the dosing schedule. While most of the RV vaccines (current and future) are administered according to a 3-dose schedule, *HRV* and *Rotavin-M1* follow a 2-dose regimen.^33^ For *HRV*, the use of a 2-dose schedule is supported by the dynamics of the immune response following natural infection.^99^ In clinical trials, a high seroconversion rate was observed after the first dose of the vaccine, whereas the second dose showed a relatively modest additional increase in the seroconversion rate, suggesting that the benefit of a second dose is limited to a catch-up effect.^99^

Of note, this immune response pattern is likely to correlate with the replication behavior of *HRV*, which is translated into vaccine antigen excretion ranging from 35% to 44%.^12^ In contrast, bovine-human reassortant vaccines were shown to have a lower replication rate compared to *HRV*.^15^ Based on available published data, bovine-human reassortant vaccines show the greatest increase in seroresponse rates after the third dose, highlighting the importance of a third dose in these vaccines.^56^ Therefore, the first dose appears to be more important in eliciting a strong immune response for the 2-dose human live-attenuated vaccine than for bovine-human reassortant vaccines.^12^ The *RV3-BB* and *116E* vaccines are given according to a 3-dose schedule, in contrast with other human live-attenuated vaccines such as *HRV* and *Rotavin-M1*. Although all clinical studies of *RV3-BB* and *116E* have been designed using the 3-dose schedule, to date, no rationale or explanation for this schedule have been published.^25,26^

Another aspect the vaccines differ on is the upper age limit of the vaccination schedule (Tables 1–Table 3). The earliest age by which the vaccination schedule can be completed is 10 weeks of age with *HRV*.^19^ This timeline is beneficial as it offers an early protection before the peak of naturally occurring RV infection.^100^

In addition, early completion of the vaccination could also limit the potential overlap between the natural IS peak and the increased risk of IS following RV vaccination.^101,102^ However, the implementation of rigid time-restrictions may pose challenges for completion of the schedule, particularly in developing countries where delays in vaccination are common. Analysis of the benefit-risk profile of RV vaccination without age restrictions suggests that in low- and middle-income countries, the additional lives saved by removing age restrictions for RV vaccination would far outnumber the potential excess of vaccine-associated IS deaths.^103,104^ As a result, and while still promoting timely vaccination, the WHO removed the recommendation of age restriction for rotavirus vaccination in 2013 in order to improve vaccine coverage. However, as most severe cases of RVGE occur earlier in life, RV vaccination of children older than 24 months is not recommended by the WHO.^2^

Vaccination scheduling is an important factor underlying compliance (adhering to the recommended immunization schedule) and completion (receiving all doses − not necessarily on schedule) of vaccination, which can greatly affect vaccine coverage. Coverage, in turn, has a substantial effect on the impact of RV vaccination, with greater reductions in the number of RV-positive samples and in RVGE hospitalizations in regions where coverage is higher.^105,106^ The *LLR* and *BRV-PV* vaccines have the broadest schedules, spread over 36 months for *LLR*, and with a maximum age of completion of 24 months for *BRV-PV*.^50^ While these extended limits may help to ensure completion of the schedule and improve coverage, they need to be carefully evaluated in terms of benefit-risk profile and real value of vaccination. Interestingly, studies carried out based on databases report higher compliance and completion for 2-dose schedule compared to 3-dose regimen.^107,108^

### 4.4 Efficacy, effectiveness and impact

A correlate of protection for RVGE would facilitate timely evaluation of vaccination strategies and the next generation of RV vaccines.^109^ There are no established correlates of protection for RV vaccines to date – only a surrogate marker of efficacy exists for *HRV*.^11,110^ Consequently, RV vaccines can only be licensed based on clinical efficacy data (***see*** Tables 1–table 3). However, several studies have identified that post-vaccination anti-RV IgA seropositivity (i.e. antibody concentration ≥20 units/mL) may serve as a useful correlate of efficacy in clinical trials on the *HRV (Rotarix)* vaccine,^109,111^ with IgA seroconversion conferring substantial protection against any severe RVGE up to the age of one year.^112^

The real-world use of the well-established *HRV* and *HBRV* vaccines has generated a great wealth of efficacy, effectiveness, and impact data. This includes data in pre-term, low birth weight infants, and other at-risk populations such as human immunodeficiency-virus (HIV)-infected or malnourished children.^13,36,38,88,95,113–120^ In contrast, to date, many of the locally marketed or recently launched vaccines have a limited record of efficacy and/or effectiveness data in global settings. Some of these vaccines with limited global experience data, namely *116E* and *BRV-PV* (both locally manufactured), have received WHO prequalification, allowing accelerated introduction of RV vaccination in high-mortality countries (with the additional support of GAVI, PATH, and UNICEF). However, collection and analysis of post-licensure data through an active surveillance system will be critical to assess the safety and effectiveness of these vaccines. In the case of local vaccines in use for several years (e.g. *LLR* in China and *Rotavin-M1* in Vietnam), the establishment and maintenance of national databases accurately recording health outcomes for RVGE following vaccine implementation would be beneficial in providing estimates of vaccine effectiveness in real-world settings and information about the safety profile of those vaccines (see safety section below).

As previously mentioned, current RV vaccines (namely *HRV* and *HBRV*) show higher efficacy in high-income countries compared to low-income countries (Tables 1–table 3). Although this phenomenon is commonly observed for oral vaccines, such as cholera or polio vaccines, the exact causes underlying this trend remain unclear.^121^ However, despite their lower efficacy, both *HRV* and *HBRV* vaccines have shown substantial real-world impact in developing countries where a high disease burden is present.^36^ Candidate RV vaccines based on non-oral approaches (e.g. parenterally administered recombinant proteins and VLPs)^25,26^ provide potential pathways into increasing vaccine efficacy in developing countries.

While not yet reported for new vaccines, immunization with *HRV* and *HBRV* has shown to provide substantial indirect benefits (community or herd protection) in some high- and middle-income countries, where the RV-related hospitalization of children too old to receive the vaccine decreased by 24%–89% upon implementation of RV immunization programs.^88,122–127^ Community protection associated with RV vaccination is most prominent in the first 3 years of a child’s life, however, children who are too young to receive the vaccine may also benefit from this protection.

In addition, there is evidence for RV vaccination having a positive impact on nosocomial infections and providing benefits with regards to health economics outcomes, particularly *HRV* and *HBRV*.^113,128–130^ More recently, the possibly positive impact of RV vaccination on type 1 diabetes and celiac disease, as well as its contribution in reducing childhood seizure hospitalization risk, were documented.^131–133^ In the context of new RV vaccines, evidence for such indirect effects should also be explored. Recent health economics analyses (cost-effectiveness evaluations) have been generated for *116E* and *BRV-PV*.^134^

### 4.5 Safety

The history of RV vaccine development has been greatly influenced by the withdrawal of the *HRRV* vaccine due to its association with IS, a naturally occurring rare event in infants mostly between 4 and 10 months of age.^18^ As the increased risk of IS following RV vaccination is very low, it was not detected in pre-licensure studies but only after marketing authorization. Following this incident, a thorough safety evaluation − especially regarding IS − was required for all second-generation RV vaccines.^135^ In addition, extensive post-marketing surveillance assessments of IS-related risks were also requested by regulatory bodies.^136^

The background incidence of IS following RV vaccination in infants <1 year of age ranges from 25 to 101 per 100,000 infants per year in developed countries (data from USA and Australia), with a mean incidence of 74 per 100,000 infant per year (data from 35 studies).^137,138^ Although the risk of IS was not identified in large pre-licensure clinical trials with *HRV* and *HBRV*,^21,22,139^ post-marketing surveillance studies have suggested the existence of a class effect for both vaccines, albeit much lower than for *HRRV*.^140–142^ According to data from observational safety studies, administration of these vaccines can result in up to six additional cases of IS per 100,000 infants, especially during the 7 days following the first dose.^141^

According to the 2018 updated WHO Global Advisory Committee on Vaccine Safety (GAVCS) report − despite the small risk of IS associated with RV vaccines − the safety profile of *HRV* and *HBRV* is acceptable, with the benefits of vaccination largely exceeding its risks. Moreover, for new vaccines such as *BRV-PV* and *116E*, the benefit-risk profile remains in favor of RV vaccination although the need for further follow-up studies on newer vaccines was highlighted in this report.^143,144^

In this context and as mentioned above (see section 3. dosing schedule), the timing of vaccine administration remains an important feature to consider. Indeed, the earlier the vaccines are administered, the lower the expected risk of IS. Schedule compliance, and ensuring that doses are given as early as possible within the recommended timeframe, are therefore key to minimizing the overall risk of IS.^101,102^ Taking this into account, implementing neonatal or early schedules (when susceptibility to IS is low) for oral, live RV vaccines could potentially decrease the risk of IS associated with RV vaccination.

Mass administration of RV vaccines has not been associated with a general increase in the incidence of IS in countries where this has been monitored.^145–148^ In addition, a non-significant decrease in IS has recently been documented during a 2-year follow-up of children who completed the RV vaccination.^146,149,150^ If this trend on the overall incidence of IS in RV-vaccinated cohorts is confirmed by additional studies, the benefit-risk profile of RV vaccination may be even more positive.

While the safety profile with regards to IS and other adverse events (including real-world data) has been characterized in detail for the well-established vaccines *HRV* and *HBRV*, there are limited real-world safety data of recently licensed and currently unlicensed RV vaccines. The phase II trials of *RV3-BB* showed a similar safety profile (including the absence of increased frequencies of fever or gastrointestinal symptoms) in the vaccine groups compared with the placebo group.^32,37^ *Rotavin-M1* demonstrated a similar adverse events profile to *HRV* in a phase I–II adaptative trial, with the most frequently reported adverse events being irritability and fever.^30^ For *LLR*, available evidence is limited to effectiveness data, and no safety data in English-language peer-reviewed scientific journals have been released so far.^41–44^ The incidence of adverse events and serious adverse events was comparable between *116E* and placebo receivers in a key phase III trial,^29,31^ and an extensive analysis of IS cases from this trial did not suggest the existence of a link between *116E* vaccination and increased IS incidence.^151^ *BRV-PV* vaccination and placebo showed comparable adverse events and serious adverse events profiles in recent trials conducted in Niger and India, in which no confirmed IS cases IS were reported.^45,49^ In addition, studies investigating the immunogenicity and safety profile of new RV vaccines, *116E* and *BRV-PV*, have revealed that these vaccines can be safely co-administered with childhood vaccines used in NIPs.^34,93^

Safety results derived from placebo-controlled trials need to be taken with caution as such studies are not powered to detect (and exclude) the potential risk of infrequent adverse events, such as IS. Large post-marketing studies and good-quality safety databases, such as the ones used to assess the safety of *HRV* and *HBRV*, are the most appropriate methods to detect rare events. Self-controlled case series are considered the gold standard to identify the risk of adverse events in a defined time frame with a very low incidence, such as IS.^152^

Safety of *HRV* and *HRBV* has also been evaluated in special populations, in which the risk of wild-type RV infection is increased. While both vaccines have an established safety profile in pre-term infants,^120^ one of the main concerns in pre-term infants and in immunocompromised children is the risk of vaccine virus shedding, which may lead to nosocomial transmission.^153^ However, delaying vaccination until hospital discharge has its own risks, and hence opinions and guidelines on the optimal timing of vaccination is such populations differ.^154,155^ Both *HRV* and *HBRV* were found to be safe for use in HIV-infected children with asymptomatic/mildly symptomatic disease (clinical stages I and II according to WHO classification)^156^ or under antiretroviral therapy, without any evidence for vaccine virus shedding.^157–159^ *HBRV* was also well tolerated in children with congenital or acquired intestinal disease requiring resection, administration was well tolerated.^160^ In infants with intestinal failure, rotavirus vaccination with *HRV* was also found to be safe and immunogenic.^161^ Data for such population is not available any of the other vaccines licensed or in development.

In this context, introducing and maintaining high-quality post-licensure surveillance systems (for both vaccine safety and circulating strains) is a fundamental point with respect to safety monitoring, especially in certain developing countries.

## 5 Conclusion

More than a decade after *HRV* and *HBRV* were licensed, the current environment of RV vaccination shows an expanding and varied landscape, with new vaccines being licensed in local markets and other vaccine candidates being in preclinical or clinical stages of development. Despite the differences in RV vaccines, there is early evidence that they may all be effective in preventing and reducing the burden of RVGE. Healthcare professionals, National Immunization Technical Advisory Groups and public health authorities will play an important role in evaluating the overall benefits of each vaccine from the perspective of individual national vaccination programs and recommending the best choice depending upon their use i.e. in private clinic or NIP use. When implementing vaccination policies, it is crucial to look beyond affordability of the vaccine and to carefully consider other aspects, such as compliance, ease of administration, ease of scheduling with other routine pediatric vaccines and safety. In addition, it is crucial for new vaccines to demonstrate similar or improved profiles compared to existing vaccines, thereby establishing a favorable safety risk profile and improving the trust of the target population toward RV immunization programs. Furthermore, considering that RV vaccination coverage is relatively low at the moment in developing countries where it is the most needed, it is essential for new vaccines to increase their accessibility and affordability. More generally, promoting compliance and completion of vaccination schedules may also key be in improving coverage and boosting the impact of vaccines. Although the path to controlling RV disease is still paved with challenges, recent advances in the field of RV vaccination offer a promising stepping stone toward this ambitious goal.

## Trademark statements

*Rotarix* is a trademark owned by or licensed to the GSK group of companies. *RotaTeq* is a trademark of Merck & Co., Inc. *Rotavac* is a trademark of Bharat Biotech. *Rotavin-M1* is a trademark of Polyvac. Lanzhou lamb rotavirus vaccine is a trademark of Lanzhou Institute of Biomedical Products. *Rotasiil* is a trademark of Serum Institute of India. *Rotashield* is a trademark of Wyeth.
